# Weighted Gene Coexpression Network Analysis Identifies Crucial Genes Involved in Coronary Atherosclerotic Heart Disease

**DOI:** 10.1155/2022/6971238

**Published:** 2022-08-02

**Authors:** Jinli Bao

**Affiliations:** Department of Internal Medicine, Municipal Hospital, Zaozhuang, Shandong 277100, China

## Abstract

**Background:**

Coronary atherosclerotic heart disease (CHD) is a lethal disease with an unstated pathogenic mechanism. Therefore, it is urgent to develop innovative strategies to ameliorate the outcome of CHD patients and explore novel biomarkers connected to the pathogenicity of CHD.

**Methods:**

The weighted gene coexpression network analysis (WGCNA) was carried out on a coronary atherosclerosis dataset GSE90074 to determine the crucial modules and hub genes for their prospective relationship to CHD. After the different modules associated with CHD have been identified, the Ontology (GO) and Kyoto Encyclopedia of Genes and Genomes (KEGG) enriched pathway analyses were conducted. The protein-protein interaction (PPI) network was thereafter performed for the critical module using STRING and Cytoscape.

**Results:**

The yellow module was recognized as the most critical module associated with CHD. The enriched pathways in the yellow module included those related to inflammatory response, positive regulation of extracellular signal-regulated kinase1/2 (ERK1/2) cascade, lipid catabolic process, cellular response to oxidative stress, apoptotic pathway, and NF-kappa B pathway. Further CytoHubba analysis revealed the top five hub genes (*MMP14*, *CD28*, *CaMK4*, *RGS1*, and *DDAH1*) associated with CHD development.

**Conclusions:**

The current study provides the prognosis, novel hub genes, and signaling pathways for treating coronary atherosclerosis. However, their potential biological roles require deeper investigation.

## 1. Introduction

Coronary atherosclerotic heart disease (CHD) is the leading cause of mortality and disability worldwide. It is characterized by coronary atherosclerosis lesions resulting from the vascular cavity stenosis, hypoxia, or necrosis [1]. Emerging evidence suggests that the main risk factors of CHD include psychological stress, lack of exercise, obesity, smoking, diabetes, dyslipidemia, and hypertension. In recent years, with rapid changes in social circumstances and improved living conditions, the morbidity associated with cardiovascular disorders has been continually increasing [2]. CHD represents the leading cause if most cardiovascular deaths. It accounts for nearly 50% of all cardiovascular disease (CAD) deaths in Western countries. The myocardial infarction resulting from CHD has been found to be one of the leading reasons for incapacity or mortality in cardiac disorder sufferers that constitute a severe hazard to the human life [3]. It has been established that individuals with CHD have a high hazard of subsequent cardiovascular disorders containing myocardial infarction (MI), stroke, and chronic heart failure (CHF) [4, 5]. With a significant increase in aging population, CHD combined with CHF has emerged as a major challenge worldwide, which can lead to utilization of excessive medical resources and aggravate patients' burden [6]. Although tremendous improvement has been achieved in the diagnostic and therapeutic strategies used for the management of CHD, many patients affected with CHD fail to get detected and assessed timely, thereby causing significant damage to their health and well-being [7, 8]. Therefore, it is urgent to decipher the exact molecular mechanism underlying CHD to develop optimal therapy.

Several algorithms based study approaches have been explored to assess the potential underlying mechanisms for the gene networks that can provide overall comprehensions into various disorders [9, 10]. Weighted gene coexpression network analysis (WGCNA) is a system biology method that could effectively differentiate gene coexpression networks of a multifaceted bioprocess to some specific modules [11]. These modules can be used to evaluate the relevant clinical features. WGCNA could explore modules of the strongly relevant genes and the correlative outer features by its particular function, acting as a valuable tool to detect potential mechanisms, prospective markers, or treatment goals for diverse diseases [12]. In recent years, WGCNA has been successfully applied in various studies on sophisticated disorders, especially in cardiovascular and cerebrovascular diseases, cancers, and neurodegenerative disorders [13–15]. Early findings on CHD mechanisms have primarily focused on pathophysiology; however, understanding of the different regulatory networks that can affect CHD remains insufficient.

In the current study, we first identified the crucial genes involved in coronary atherosclerotic heart disease by weighted gene coexpression network analysis. The enriched signaling pathways were further explored. We carried out a WGCNA analysis with the CHD-related dataset GSE90074. The crucial modules were confirmed, and an enriched pathway analysis for the crucial module was conducted to investigate the prospective biological functions. PPI networks were also constructed using the STRING and Cytoscape software.

## 2. Methods

### 2.1. Dataset Information

The coronary atherosclerosis dataset, GSE90074, was downloaded from the NCBI Gene Expression Omnibus (GEO; https://www.ncbi.nlm.nih.gov/geo/). The dataset was processed using background amendment through the R package (version 3.12) and quantile normalization with the R package (version 1.17.1) software. A total of 13080 mRNAs (including 143 samples), which contained appropriate expression data, were obtained for further investigation. The hclust (R base function) was employed to conduct a hierarchical clustering analysis, and all the outliers were omitted. Finally, the top 3000 critical genes were selected for WGCNA analysis.

### 2.2. WGCNA

GSE90074 sample data were pretreated and standardized by the WGCNA R package. Thereafter, the genes with the highest 25% variance were chosen. The soft threshold power of 5 (scale-free*R* = 0.95) and a modulesize ≥ 30were used to mine the different crucial modules. The correlation analysis between the various modules and the clinical trait was conducted by using the correlation analysis, and adj *p* < 0.01 was considered as significant. The topological overlap measure (TOM) was used to assess the network's connectivity property. A clustering dendrogram was constructed using average linkage hierarchical clustering based on the TOM matrix.

### 2.3. Identification of CHD-Related Modules Corresponding to Clinical Traits

The potential relationship between the different modules and clinical traits was examined to explore the critical modules, which could be drastically associated with the sample traits. We estimated the relevance between module eigengenes (MEs) that can generalize the expression profiles of these modules. The association was evaluated by the R statistical package WGCNA. The modules with high significance (*p* < 0.05) were employed for the hub gene selection. The connection values were exhibited in a heatmap. The modules, which were markedly connected to CHD, were selected as the key modules. We estimated the connection between ME and clinical traits and examined the clinical importance of these crucial modules.

### 2.4. Functional Enrichment Analysis

Gene Ontology (GO) and Kyoto Encyclopedia of Genes and Genomes (KEGG) pathway enrichment analyses were conducted to assess the biological significance of the various genes in the key modules by the cluster profile R package (version 3.12). GO pathway analyses were conducted for the differentially expressed genes (DEGs) using R and DAVID online software. KEGG was performed to assess the critical biological functions and pathways of the various DEGs. Adj *p* value < 0.05 was selected for the significance threshold.

### 2.5. Identification of Hub Genes

The top 20% of crucial genes were defined as potential hub genes in the crucial modules with the maximum connectivity. STRING3 database and Cytoscape v3.7.0 software were employed for the coexpression network construction. The maximal clique centrality (MCC) algorithm was used to identify the top five hub genes with the highest connectivity from the key modules.

### 2.6. Statistical Analysis

The package R was employed to assess the gene expression. The “limma” package was applied to evaluate the correction differences. The “cluster profile” package was used for functional enrichment analysis of the DEGs. The diagnostic value of the hub genes was explored by using ROC curve in SPSS 22.0. *p* < 0.05 was considered as statistically significant.

## 3. Results

### 3.1. Creation of Weighted Gene Coexpression Network

**Figure 1:**
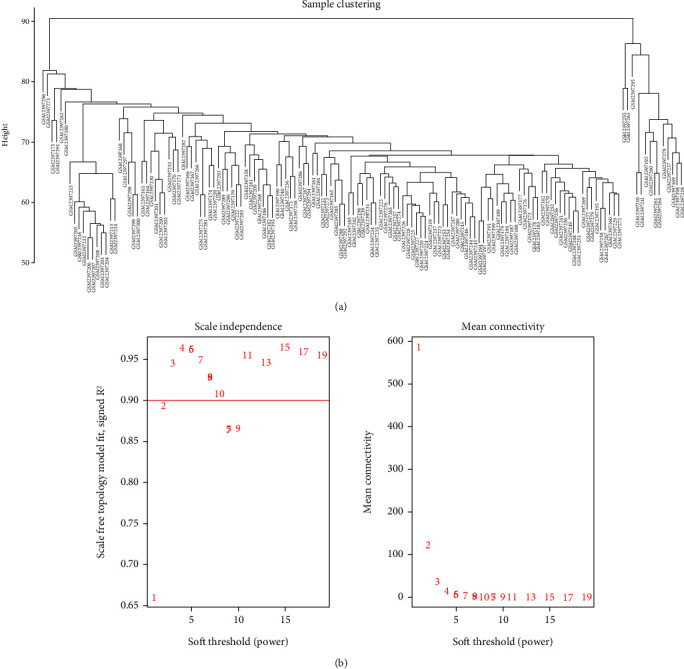
Creation of weighted gene coexpression network. (a) A sample cluster dendrogram. (b) The soft thresholding powers for the scale-free network.

The top 3000 genes based on the GSE90074 dataset were used to create the coexpression network. The clustering analysis based on the profile of sample expression values was performed to assess the possible outliers of samples (Figure 1(a)). We further evaluated the threshold by employing the scale-free topology criterion. After identifying the soft threshold, a weighted gene coexpression network was set up based on the various identified genes. The scale-free network was imported into the network topology with a soft threshold set at 5 (scale-free *R* = 0.95) (Figure 1(b)).

### 3.2. Identification of Key Modules

**Figure 2:**
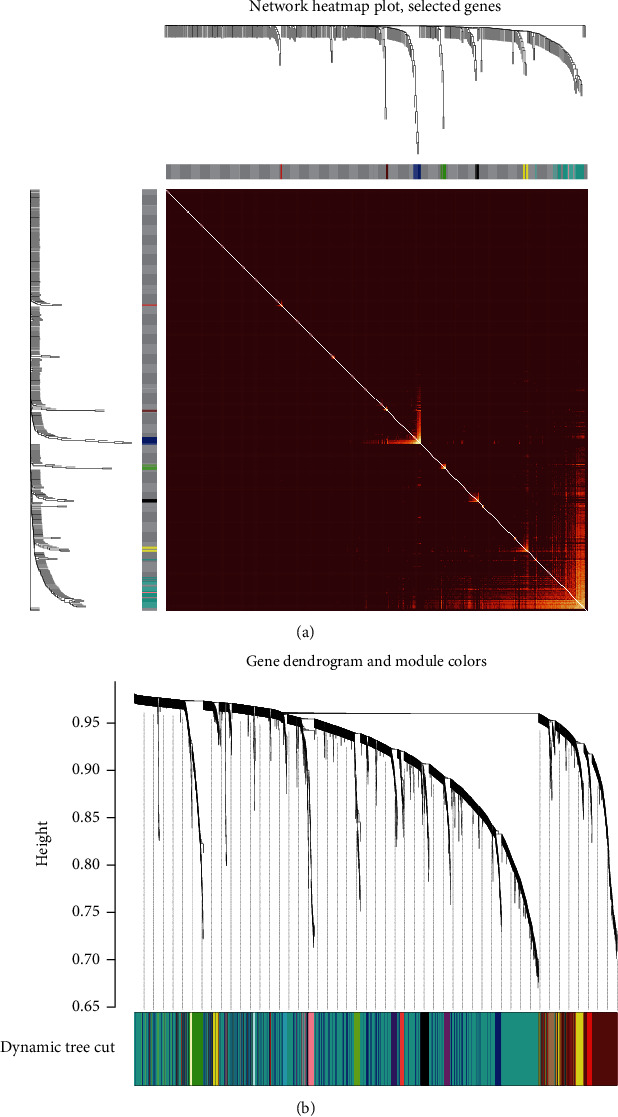
Evaluation of the key modules. (a) Cluster diagram of the various gene modules. (b) The TOM heatmap plot.

A hierarchical clustering tree was generated to mine the coexpression modules with a dynamic cut approach. The smallest quantity of the genes in each module was minModule size = 30. To construct a topological overlap matrix (TOM), the contiguous and connection matrices for the gene expression profile were analyzed. A final gene clustering tree based on the gene-gene non-*ω* similarity was set up. In addition, all the similar expression modules were merged, and nine different modules were identified. The correlation between the module genes has been demonstrated in Figure 2(a). Moreover, the different modules have been indicated by diverse colors. The TOM was visible with a heatmap that depicted adjacencies or topological overlaps (Figure 2(b)).

### 3.3. The Correlation Analysis between Modules

**Figure 3:**
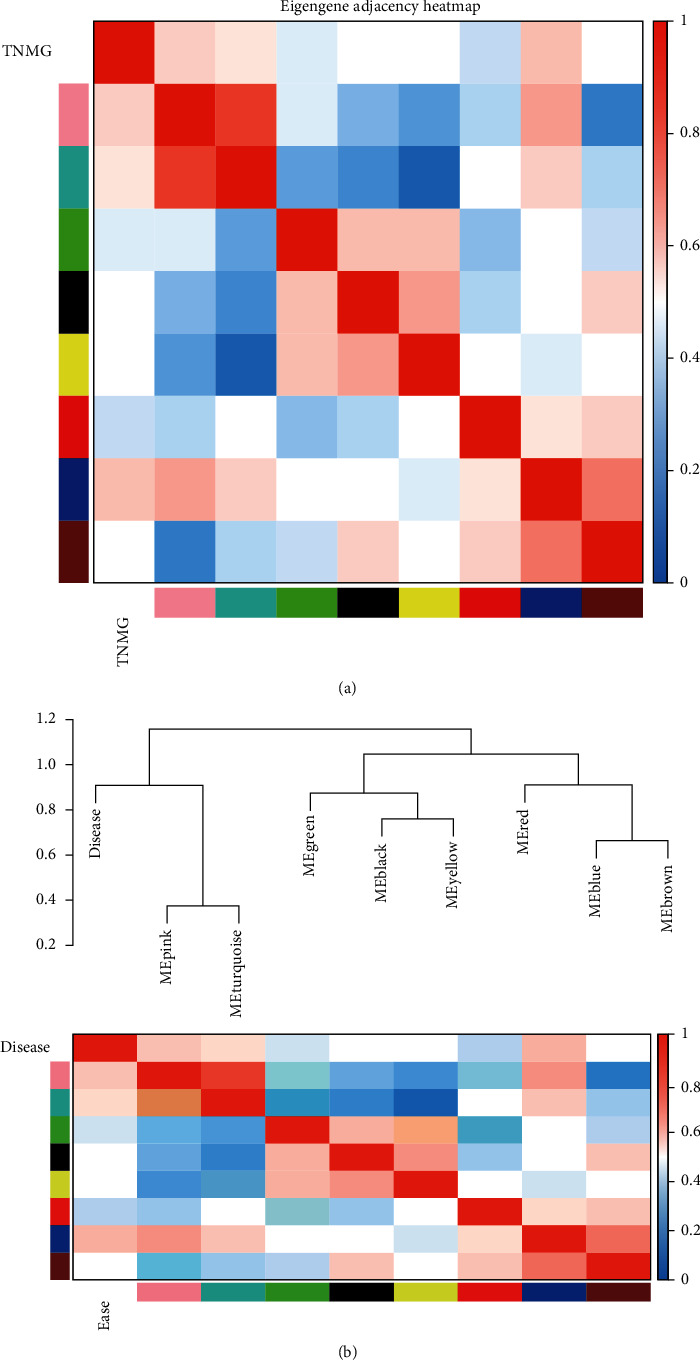
The correlation analysis between the different modules. (a) Module-trait relationship. (b) Hierarchical clustering analysis for the modules.

We further estimated the correlation between the modules to explore the potential relationship between the identified modules. The heatmap of the coexpression modules has been demonstrated in Figure 3(a). Thereafter, the clustering dendrogram for WGCNA coexpression modules was constructed to determine the similarity between the modules. The coexpression modules from WGCNA were evaluated by using hierarchical cluster analysis, and the modules in the same category exhibited similar gene expression trends (Figure 3(b)).

### 3.4. Construction of Module-Trait Relationships

**Figure 4:**
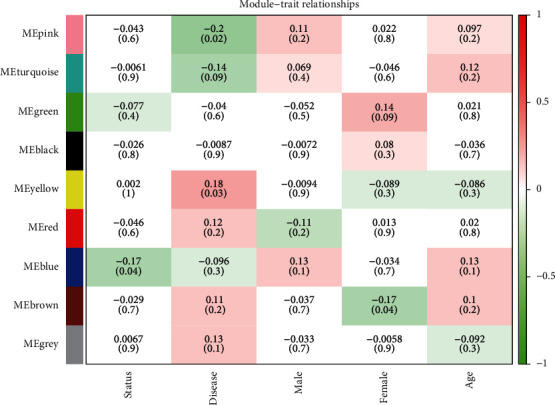
The creation of module-trait relations. The association between the modules and the clinical traits. Each row relates to a specific module, and the column corresponds to the different clinical traits.

To obtain the possible gene modules that were tightly associated with CHD, the related clinical information of the sample was mined, and the association between the nine distinctive modules and the clinical characters was evaluated. The related sample traits (survival status, disease, male, female, and age) were obtained according to the data from the GSE90074. The correlation between the traits and modules has been demonstrated in Figure 4. As determined from the evaluation of module-trait relationships, the yellow module exhibited a greater significance related to CHD and exhibited a positive relationship. The correlation coefficient was 0.18, thereby implying that the various genes from this module were positively affected by CHD progression. The pink module was observed to be negatively related to CHD progression. Furthermore, the correlation coefficient between the blue module and the survival status was −0.17, thus implying that the different genes in the blue module might be negatively connected to the disease status.

### 3.5. Correlation between the Different Modules and Clinical Traits

**Figure 5:**
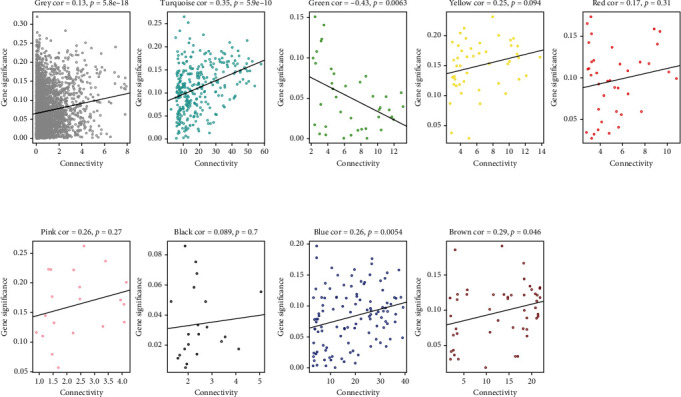
Connection between the different modules of interest and the clinical traits in the yellow module.

The module-trait association was examined by connecting the modules with sample traits to detect the apparent connotations. For clinical status trait (CHD), the brown module showed the highest positive correlation (*r* = 0.29; *p* < 0.05), but the green module (*r* = −0.43; *p* < 0.05) demonstrated the negative correlation (Figure 5). These results suggested that the brown and green modules have been recognized as the crucial modules for CHD.

### 3.6. Enrichment Analysis

**Figure 6:**
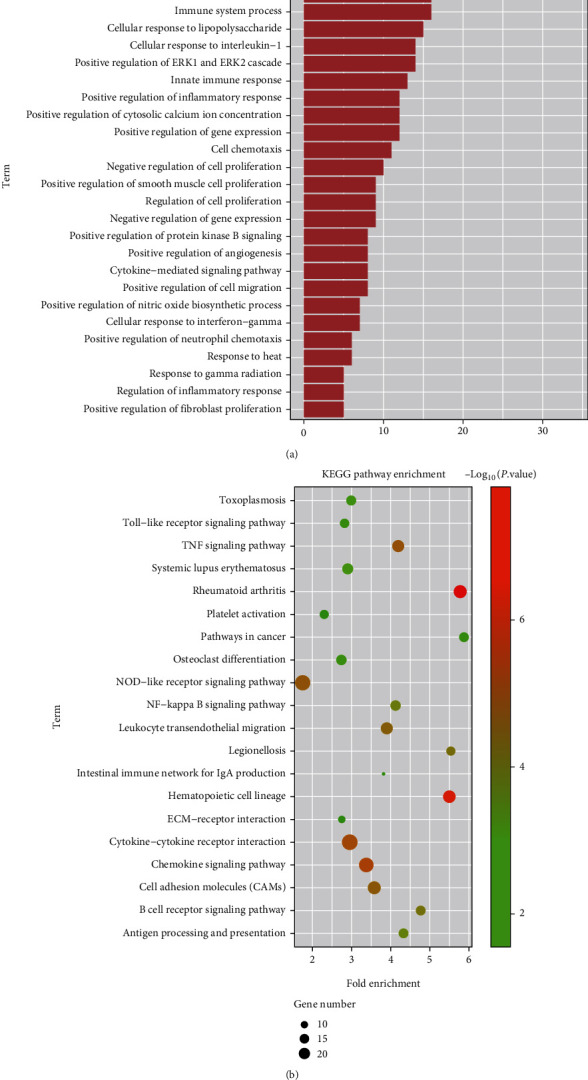
Functional enrichment analysis of the various genes in the yellow module. (a) GO, Gene Ontology. (b) KEGG, Kyoto Encyclopedia of Genes and Genomes.

According to the correlation analysis and the different clinical features, the yellow module displayed the closest association with the progression of CHD. To investigate the biological functions of the identified genes implicated in this module, 325 genes were obtained for the yellow module. GO and KEGG enrichment analyses were carried out. Regarding the GO pathway analysis, it was observed that the genes in the yellow module were enriched in the positive regulation of angiogenesis, smooth muscle cell proliferation, inflammatory response, ERK1 and ERK2 cascade, cytosolic calcium ion concentration, and cytokine-mediated signaling pathway (Figure 6(a)). For the KEGG enrichment analysis, the various genes in the yellow module were primarily enriched in the inflammatory response, innate immune response, lipid catabolic process, cellular response to oxidative stress, apoptotic signaling pathway, NF-kappa B signaling pathway, and NOD-like receptor signaling pathway (Figure 6(b)).

### 3.7. Identification of the Hub Genes

**Figure 7:**
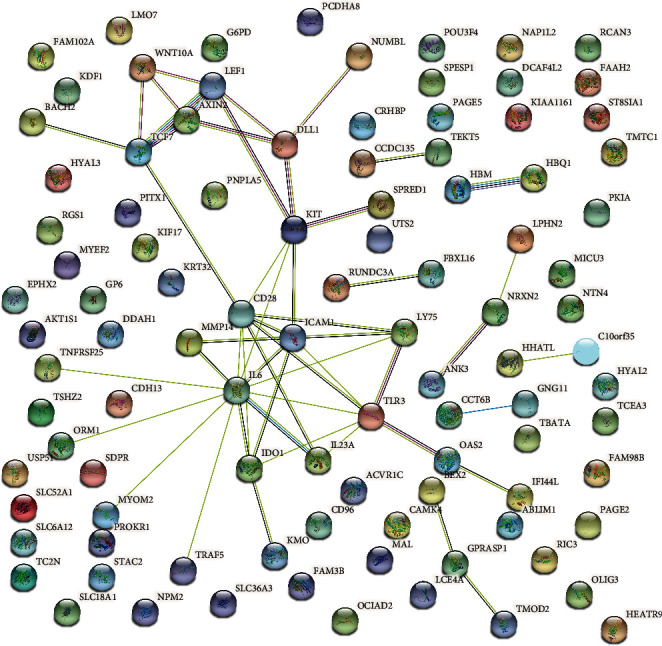
Construction of PPI network related to the yellow module. The hub genes with the highest connectivity in the yellow module.

We used the STRING online tool to obtain a PPI network to determine the various hub genes that can effectively modulate the progression of CHS. The hub genes of the yellow module were introduced into the STRING database for PPI exploration, and the various networks were created in Cytoscape (Figure 7). The top five hub genes with the highest MCC scores that were identified in the yellow module were *MMP14*, *CD28*, *CaMK4*, *RGS1*, and *DDAH1*.

### 3.8. Validation of the Various Hub Genes

**Figure 8:**
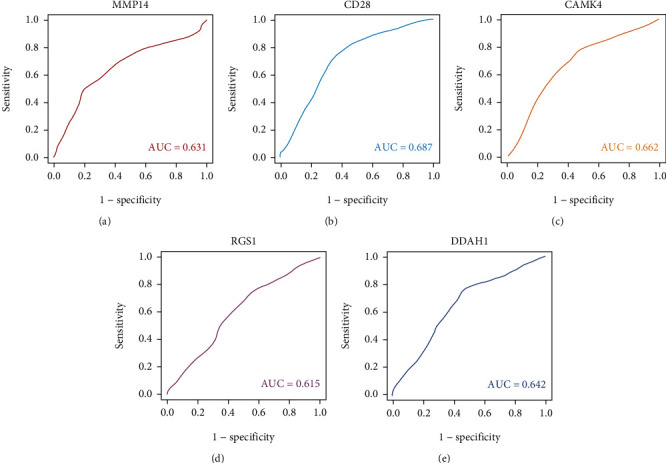
ROC curves for the hub genes. ROC: receiver operating characteristic; AUC: area under curve.

The underlying clinical importance of the various hub genes was also explored by ROC analysis. All the five genes exhibited prospective diagnostic values (*p* < 0.05) and ROC curves of these hub genes have been depicted in Figure 8.

## 4. Discussion

Coronary atherosclerotic heart disease is one of the most commonly diagnosed cardiac diseases in the world [16]. It usually results from the congestion of the coronary artery by atherosclerotic plaque. In many industrialized countries, CHD has become the leading cause of mortality among adults, accounting for 30.8%~40% of deaths globally, which can severely endangered the well-being and survival of human beings [17]. With the rapid development of our economy and the acceleration of population aging worldwide, the morbidity associated with CHD has been also significantly increasing worldwide. Social and economic development has made the average life expectancy of human beings significantly longer than in the past, and the world is aging. With the aggravation of population aging, the incidence rate of chronic diseases such as CHD is on the rise. The prevention and cure of CHD are facing severe problems and challenges. Therefore, there is an urgent need to investigate the underlying molecular mechanisms to develop novel therapeutic strategies for CHD.

With the innovation and development of DNA sequencing and chip technology, bioinformatics methods have been extensively employed for research related to the various chronic diseases, such as non-small-cell lung cancer [18], gastric cancer [19], sepsis [20], pressure hydrocephalus [21], amyotrophic lateral sclerosis [22], schizophrenia [23], hypertension [24], hypertrophic cardiomyopathy [25], and pulmonary artery high-pressure disease [26]. In the current study, we have used multiple bioinformatics methods to determine the potential biological mechanisms of CHD. GEO database is a common gene chip database established by NCBI. The weighted gene coexpression network analysis (WGCNA) algorithm is a novel biological technique that provides the association method between the various gene modules and clinical traits to explore the genes involved in specific phenotypical traits. It has been found that compared with the classical difference gene expression analysis, which primarily focuses about the genes illustrating the variance between the different groups, WGCNA clusters can provide information about the coexpressed genes in an unbiased manner into modules that could be associated with clinical traits. This study employed WGCNA to detect the crucial modules implicated in CHD development. WGCNA was performed on the dataset GSE90074, which comprised of coronary atherosclerotic heart disease samples. Among the nine coexpression modules acquired by WGCNA, the yellow module was mainly associated with CHD development. We recognized that the yellow module could serve as a critical module related to CHD progression. We then conducted a functional enrichment investigation to explore the diverse biological functions of the genes implicated in this module. We further investigated hub genes by constructing a PPI network to explore the crucial genes involved in CHD development.

The enrichment analysis of the yellow module's biological function and pathway illustrated that the genes were primarily enriched in the inflammatory response, innate immune response, lipid catabolic process, cellular response to oxidative stress, and apoptotic signaling pathway, which was concurrent with the previous findings. For instance, Libby [27] demonstrated that the process of CHD might be considered as an inflammatory response to coronary artery injury. He revealed that the varying pathological conditions could cause immune responses that could accelerate the transmigrating of monocyte adhesion into the subintimal space. Moreover, another study has highlighted the critical role of lipid accumulation in the processes linked to onset and subsequent destruction of atherosclerotic plaques. They depict that these lipid species could be considered the basis for the forecast of CHD [28]. Kibel et al. reported that chronic inflammation was the crucial pathophysiologic process underlying atherosclerosis, and oxidative stress can play a vital role in regulating vascular homeostasis. It has been established that imbalance in the oxidant/antioxidant ratio could result in oxidative stress and substantial vascular damage [29]. In addition, a recent study has highlighted that macrophage foam cells can increase the inflammatory response and accelerate the eventual complications of atherosclerosis [30]. Zakynthinos and Pappa indicated that inflammation was the major cause of plaque rupture and can promote acute atherothrombotic vascular occlusion and infarction [31]. Another study reported that ROS can induce the activation of the NF-*κ*B pathway which in turn can effectively promote the development of coronary calcification [32]. Our results also confirmed the involvement of the inflammatory response, innate immune response, lipid catabolic process, cellular response to oxidative stress, and metabolism-related processes in CHD.

The various hub genes were identified by constructing a PPI network for the genes from the yellow module by the STRING and Cytoscape. Eighty-three essential genes were eventually confirmed for the yellow module. The top five identified genes with the highest MCC scores were *MMP14*, *CD28*, *CaMK4*, *RGS1*, and *DDAH1*. It has been reported previously that the matrix metalloproteases (MMPs) are a critical family of proteins implicated in various biological processes, including angiogenesis, vascular repairing, and inflammatory response. The roles of few MMPs have been also implicated in CAD in which the specific proteases appear to exhibit differential effects. Among these, the expression of MMP14 was found to be significantly enhanced in injury-spawned blood vessels [33]. Schmitt et al. further demonstrated that the ratio of MMP-14/TIMP-2 in aortic tissues could regulate the activation of Pro-MMP-2 in ascending thoracic aortic aneurysms [34]. Moreover, MMP-14 has been found to play a crucial role in the development of coronary vascular lesions [35]. Burton et al. reported that MMP14 can function as an important regulator gene related to the progression of atherosclerosis and vascular calcification [36]. CD28 is a homodimeric cell surface receptor that is predominantly expressed on T-cells. For years, the role of CD28^−^ T cells has been implicated in numerous inflammatory disorders. Sun et al. found that CD4(+) CD28(-) T cells were detected in atherosclerotic plaques and the peripheral circulation blood in patients with acute coronary syndrome and exerted critical roles in plaque ruptures [37]. Accumulating evidence has also indicated that patients with CD28^−^ T cell extensions can exhibit atherosclerotic variations [38]. Furthermore, CD28^−^ T cells are found to be upregulated in the clinical situation linked to acute coronary syndrome. The Ca2^+^-dependent protein kinase IV (CaMK4), which acts as a crucial member of the CaMK family, is a serine/threonine kinase that plays a multifunctional role in modulating the immune response. Emerging evidence has suggested that CaMK4 can exert a pivotal role in the progression of Th17 cells and in regulating IL-17 production by Th17 cells [39]. In addition, Ichinose et al. suggested that CaMK4 suppression could result in the significant reduction of IFN-*γ* production in T and B cells [40]. Overall, consensus derived from the various studies indicate that coronary atherosclerosis is primarily an inflammatory disease of the coronary artery. Santulli et al. suggested that CaMKIV can play an essential role in blood pressure modulation by controlling the endothelial nitric oxide synthase activity [41]. They further reported that the loss of CaMK4 can markedly accelerate hypertension progression, accompanied by endothelial dysfunction and organ injury [41]. As a marker of endothelial dysfunction, asymmetric dimethylarginines (ADMAs) have been recognized as the hazard elements for diverse cardiovascular conditions such as pulmonary hypertension, coronary heart disease, and portal hypertension [42, 43]. Dimethylarginine dimethylaminohydrolase-1 (DDAH1) can play a critical role in the decomposition process of ADMA and NO signaling. A large body of evidence has indicated that the loss of function *DDAH1* can directly relate to the rising morbidity of the coronary heart disease, thrombosis, and stroke. In addition, DDAH1 has been considered a crucial biomarker and can display strong prognostic value in patients with CHD [44]. Recently, several studies have suggested that DDAH1 level was correlated with amplified risk of stroke and CHD [45]. Regulator of G-Protein Signalling-1 (RGS1) is the prototype of a seven-transmembrane receptor fused with an RGS domain that can deactivate G-protein signaling and significantly reduces the response to the sustained chemokine stimulation [46]. RGS1 has been identified to display differential expression in CHD by whole-genome expression arrays [47]. Patel et al. demonstrated that RGS1 could effectively function as a negative regulator in various chronic inflammatory diseases [48]. Consistent with these reports, our results illustrated that *MMP14*, *CD28*, *CaMK4*, *RGS1*, and *DDAH1* played critical roles in the progression of CHD. Our ROC analysis also revealed the potential clinical importance of the five hub genes, and it was found that all these five identified genes exhibited prospective diagnostic values in CHD.

Taken together, WGCNA was carried out on a CHD dataset. The yellow module was found to be the most crucial module for CHD progression among the nine modules. The yellow module was confirmed to be related to inflammatory response, innate immune response, lipid catabolic process, cellular response to oxidative stress, and apoptotic signaling pathway. We also revealed that five crucial genes (*MMP14*, *CD28*, *CaMK4*, *RGS1*, and *DDAH1*) played significant roles in pathophysiological mechanisms of CHD. Our findings might facilitate the forthcoming experimental studies aimed at exploring the functions of the critical genes involved in CHD. In addition, the hub genes obtained might provide novel strategies for targeted therapy of CHD and contribute to achieving an enhanced comprehension of the underlying mechanisms of CHD. However, this study still has some limitations. Although the hub genes were generated from a different dataset, it still needs to be further validated in a larger patient cohort.

## Data Availability

The labeled dataset used to support the findings of this study are available from the corresponding author upon request.
